# Insidious Intrahepatic Portal Vein Embolism Due to Superior Mesenteric Vein Aneurysm

**DOI:** 10.7759/cureus.95496

**Published:** 2025-10-27

**Authors:** Tohru Nakajima, Hiroshi Yasuhara, Kouichi Taniwaka

**Affiliations:** 1 Department of Surgery, Kyoritsu Kanbara General Hospital, Shizuoka, JPN; 2 Surgical Center, University of Tokyo Hospital, Tokyo, JPN

**Keywords:** embolism, intrahepatic portal vein, intraluminal thrombus, liver dysfunction, rupture, superior mesenteric vein aneurysm

## Abstract

There is limited information on the natural history of superior mesenteric vein (SMV) aneurysms. SMV aneurysms have the risk of aneurysm rupture due to enlargement and liver dysfunction caused by recurrent embolism. There have been a few reports of SMV aneurysms, but no clear guidelines exist regarding the management of SMV aneurysms. We report a case associated with ‘silent’ embolism of the intrahepatic portal vein without any clinical symptoms, which could be a part of its natural history. Accumulation of more cases is needed to more accurately understand the natural history of this rare disease.

## Introduction

There is limited information on the natural history of visceral aneurysms [1-5] and even less on superior mesenteric vein (SMV) aneurysms, due to the rarity of reported cases [6-8]. Most case reports concern severe clinical conditions such as rupture or gastrointestinal bleeding, which often lead to the diagnosis.

Fulcher et al. described in detail a case of symptomatic portal vein aneurysm (associated with SMV aneurysm), suggesting that portal hypertension might be a factor related to rupture and gastrointestinal bleeding [9]. However, they did not mention portal hypertension or liver damage caused by recurrent emboli, whose source can be an SMV aneurysm. Some other reported cases have been found incidentally during a medical checkup or an examination for accompanying diseases [10]. Some reports indicated that the majority of SMV aneurysms caused no complications and could be managed with follow-up ultrasound examinations [3-5,7]. We report a case associated with ‘silent’ embolism of the intrahepatic portal vein (iHPV) without any clinical symptoms, which could be a part of the natural history of this condition.

## Case presentation

**Figure 1:**
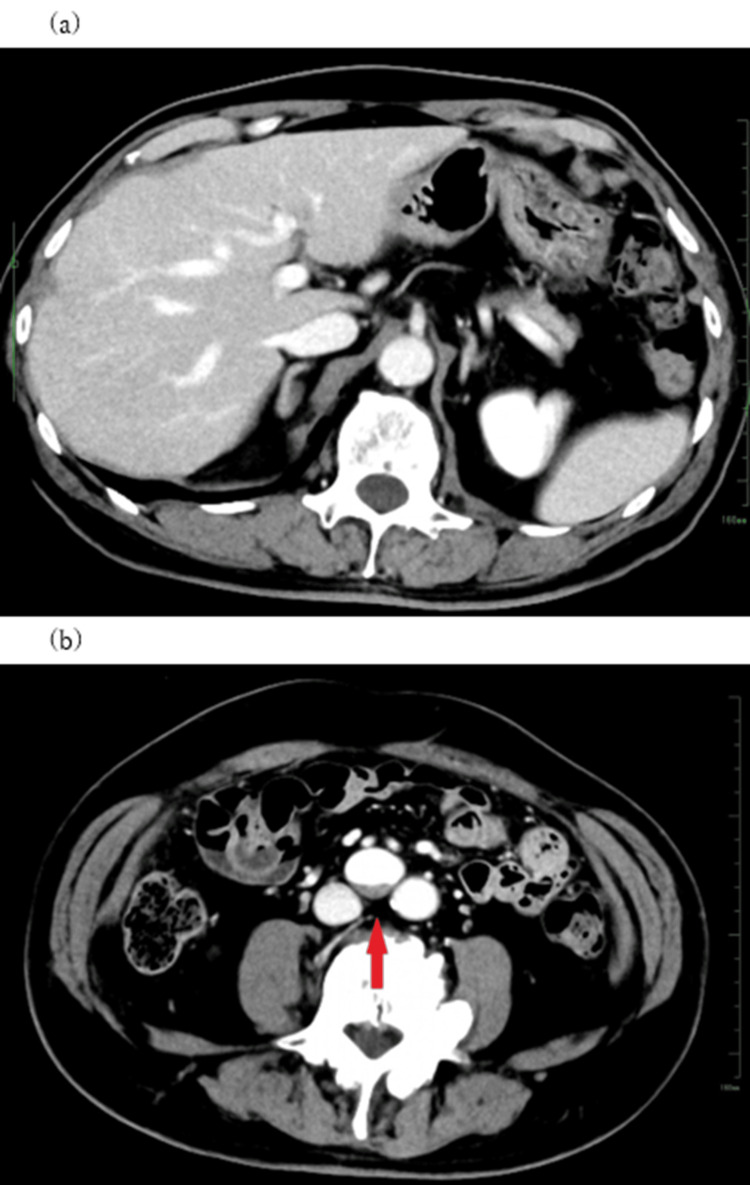
Axial view of the enhanced abdominal CT at first detailed examination. (a) No embolus of the intrahepatic portal vein is revealed. (b) Intraluminal thrombus is visualized in the superior mesenteric vein (SMV) aneurysm (arrow).

A 78-year-old man underwent a routine comprehensive medical checkup in February 2023. Slight enlargement of the bilateral common iliac arteries was found in the abdominal echogram, and he underwent a detailed abdominal CT examination one month later. The CT images revealed an SMV aneurysm, which was dilated to approximately 25 mm with intraluminal thrombi, although there was no remarkable enlargement of the common iliac artery (Figure 1b). No thrombus was visible in the iHPV (Figure 1a).

**Figure 2:**
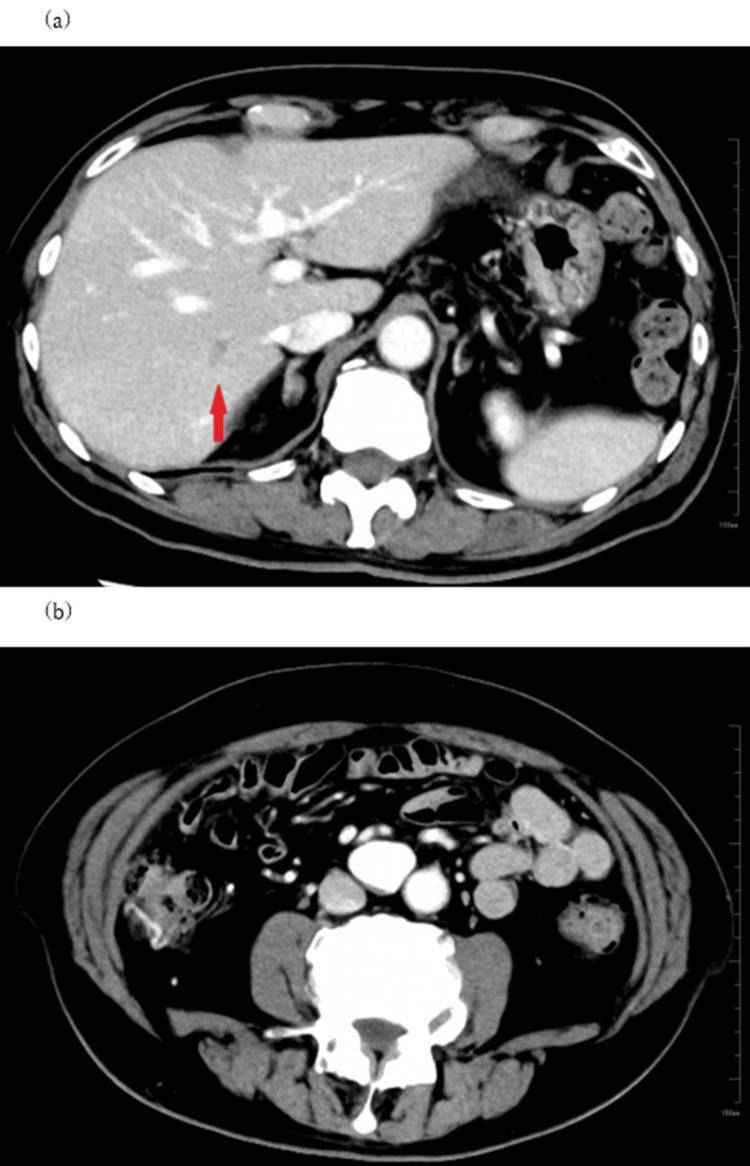
Axial view of the enhanced abdominal CT after one year. Intraluminal thrombus in the superior mesenteric vein (SMV) aneurysm has disappeared at this point (a), while an embolus of the intrahepatic portal vein (arrow) is revealed (b).

Follow-up CT examination carried out one year later revealed that the intraluminal thrombus in the SMV aneurysm had disappeared (Figure 2a), while thrombus had become visible in the iHPV (Figure 2b).

At that time, he was in good physical condition, and the laboratory test results of liver function were within normal limits (Table 1).

**Table 1:** Laboratory data at initial consultation. AST: aspartate aminotransfenase, ALT: alanine aminotransferase, LD: lactate dehydrogenase, ALP: alkaline phosphatase, γGTP: gamma glutamyltransfenase, BUN: blood urea nitrogen, eGFR: estimated glomerular filtration rate

Labolatory data	Result	Reference range	Unit of measurement
Blood count			
White blood cell	3100	3300-8600	/μL
Red blood cell	469	435-55	×10⁴/μL
Hemoglobin	15.5	13.7-16.8	g/dL
Platelet	9.1	15.8-34.8	×10⁴/μL
Chemistry			
AST	22	13-30	U/L
ALT	22	10-42	U/L
LD	187	124-222	U/L
ALP	69	38-113	U/L
γGTP	30	13-64	U/L
Cholinesterase	269	240-486	U/L
Total bilirubin	0.7	0.2-1.0	mg/dL
Total protein	7.1	6.6-8.1	g/dL
Albumin	3.9	4.1-5.1	g/dL
C-reactive protein	0.09	0.00-0.14	mg/dL
BUN	23.4	8.0-20.0	mg/dL
Creatinine	1.14	0.65-1.07	mg/dL
eGFR	48.5	60.0-125.0	mL/min
Sodium	138.6	138-145	mmol/L
Potassium	4.29	3.6-4.8	mmol/L
Chloride	102.5	101-108	mmol/L

**Figure 3:**
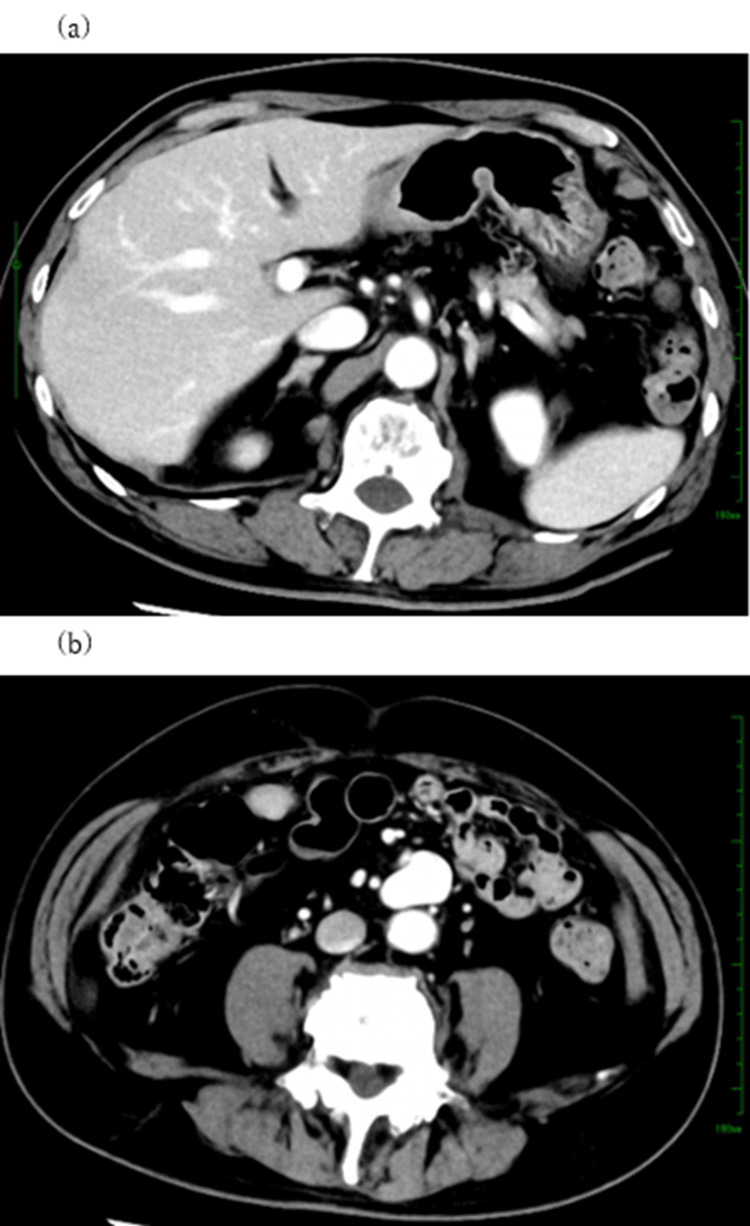
Axial view of the enhanced abdominal CT after one year and nine months. Both an embolus of the intrahepatic portal vein (a) and intraluminal thrombus in the superior mesenteric vein (SMV) aneurysm (b) have disappeared.

Considering his age and wishes, we decided to follow up with observation. Nine months later, he underwent a follow-up CT examination, which did not visualize the preexisting thrombi in either the iHPV or SMV aneurysm. There was no remarkable change in the size of the SMV aneurysm (Figures 3a-3b).

He had a history of hypertension and had been prescribed an antihypertensive agent for about seven years before his first visit. He had no history of habitual smoking. His physical status was otherwise normal. He had never experienced signs or symptoms associated with intestinal bleeding or rupture of the aneurysm throughout his clinical course.

## Discussion

SMV aneurysm is an infrequent clinical condition. Few previous reports have detailed descriptions of their natural history and clinical features. Sfyroeras et al. [3] described that there were two main theories regarding the aetiology of portal vein aneurysm: congenital and acquired. Congenital portal venous aneurysms are generally considered stable lesions, and regular follow-up is usually sufficient. Acquired portal venous aneurysms, mainly when they are combined with liver cirrhosis and portal hypertension, can have a more unpredictable evolution through time, requiring closer follow-up and intervention when complications occur [3]. Smith et al. [7] reported the first case of SMV aneurysm rupture. Ninety-four percent of visceral venous aneurysms (VVAs) caused no complications and were stable in size on follow-up ultrasound. However, previous asymptomatic VVA ruptures have the lethal potential [7]. Nitesh et al. concluded that, even though the reported incidence of the rupture is very low, this finding signifies the unpredictability in the outcomes of SMV aneurysms when stratified based on size criteria alone [5]. Wolosker et al. reported that some patients with SMV aneurysm have vague abdominal pain, which is usually recurrent and localized in the right upper quadrant [6]. Our findings suggested that this type of abdominal pain, if present, might be caused by recurrent emboli of the iHPV due to SMV aneurysm thrombosis. The fact that the iHPV embolism disappeared over time in our case, likely through a thrombolytic process, may support our speculation. To our knowledge, no reports have presented a potential relationship between iHPV embolism and its clinical symptoms in patients with SMV aneurysm.

Late complication is another crucial issue of SMV aneurysm, as well as acute complications, such as rupture or bleeding. Our patient was completely unaware of any abdominal symptoms, and his laboratory data showed no abnormality of liver function or coagulation, even though the CT scan showed an embolus of the iHPV due to SMV aneurysm thrombosis. Thus, we had very little chance of detecting iHPV emboli by routine laboratory tests or diagnostic imaging since the emboli often remain in the portal vein for a short period due to the thrombolytic process. Nevertheless, our case may shed light on its natural history. Repeated emboli might occur and aggregate to cause liver damage insidiously [11]. Only the accumulation of further reported cases could elucidate the more accurate natural history of this rare condition.

## Conclusions

SMV aneurysm may have a risk of liver dysfunction due to recurrent embolism of the iHPV, as well as a risk of rupture. However, the cause of these complications and their long-term clinical course are not clear at present. Accumulation of more cases is needed to understand the more accurate natural history of this rare disease.
